# Efficiency of High and Standard *b* Value Diffusion-Weighted Magnetic Resonance Imaging in Grading of Gliomas

**DOI:** 10.1155/2020/6942406

**Published:** 2020-09-14

**Authors:** Mansour Al-Agha, Khaled Abushab, Khetam Quffa, Samy Al-Agha, Yasser Alajerami, Mohammed Tabash

**Affiliations:** ^1^Radiology Department, Al-Shifa Medical Complex, Ministry of Health, Gaza, State of Palestine; ^2^Medical Imaging Department, Faculty of Applied Medical Sciences, Al Azhar University-Gaza, Gaza, State of Palestine

## Abstract

**Background:**

Glioma is the most common fatal malignant tumor of the CNS. Early detection of glioma grades based on diffusion-weighted imaging (DWI) properties is considered one of the most recent noninvasive promising tools in the assessment of glioma grade and could be helpful in monitoring patient prognosis and response to therapy.

**Aim:**

This study aimed to investigate the accuracy of DWI at both standard and high *b* values (*b* = 1000 s/mm^2^ and *b* = 3000 s/mm^2^) to distinguish high-grade glioma (HGG) from low-grade glioma (LGG) in clinical practice based on histopathological results.

**Materials and Methods:**

Twenty-three patients with glioma had DWI at l.5 T MR using two different *b* values (*b* = 1000 s/mm^2^ and *b* = 3000 s/mm^2^) at Al-Shifa Medical Complex after obtaining ethical and administrative approvals, and data were collected from March 2019 to March 2020. Minimum, maximum, and mean of apparent diffusion coefficient (ADC) values were measured through drawing region of interest (ROI) on a solid part at ADC maps. Data were analyzed by using the MedCalc analysis program, version 19.0.4, receiver operating characteristic (ROC) curve analysis was done, and optimal cutoff values for grading gliomas were determined. Sensitivity and specificity were also calculated.

**Results:**

The obtained results showed the ADC_mean_, ADC_ratio_, ADC_max_, and ADC_min_ were performed to differentiate between LGG and HGG at both standard and high *b* values. Moreover, ADC values were inversely proportional to glioma grade, and these differences are more obvious at high *b* value. Minimum ADC values using standard *b* value were 1.13 ± 0.17 × 10^−3^ mm^2^/s, 0.89 ± 0.85 × 10^−3^ mm^2^/s, and 0.82 ± 0.17 × 10^−3^ mm^2^/s for grades II, III, and IV, respectively. Concerning high *b* value, ADC_min_ values were 0.76 ± 0.07 × 10^−3^ mm^2^/s, 0.61 ± 0.01 × 10^−3^ mm^2^/s, and 0.48 ± 0.07 × 10^−3^ mm^2^/s for grades II, III, and IV, respectively. ADC values were inversely correlated with results of glioma grades, and the correlation was stronger at ADC_3000_ (*r* = −0.722, *P* ≤ 0.001). The ADC_3000_ achieved the highest diagnostic accuracy with an area under the curve (AUC) of 0.618, 100% sensitivity, 85.7% specificity, and 85.7% accuracy for glioma grading at a cutoff point of ≤0.618 × 10^−3^ mm^2^/s. The high *b* value showed stronger agreement with histopathology compared with standard *b* value results (*k* = 0.89 and 0.79), respectively.

**Conclusion:**

The ADC values decrease with an increase in tumor cellularity. Meanwhile, high *b* value provides better tissue contrast by reflecting more tissue diffusivity. Therefore, ADC-derived parameters at high *b* value are more useful in the grading of glioma than those obtained at standard *b* value. They might be a better surrogate imaging sequence in the preoperative evaluation of gliomas.

## 1. Introduction

Gliomas are one of the most life-threatening malignant types of central nervous system (CNS) tumors and remain the most difficult cancer to manage and treat [1]. They have an annual incidence rate of about 5 in 100,000 in the United States and represent 4.9% of all cancer cases in Palestine [2, 3]. Glioma is divided into four grades, and the most aggressive grade is glioblastoma multiform (grade IV), which accounts for 47% of malignant CNS tumors, and its prognosis is the worst among all cancers with five years' survival rate of merely 5.5% [4]. Over the past few years, MRI became popular in clinical use. It frequently detects and provides high-resolution accuracy in tumor border delineation, maximizing the resection of the tumor, and increases the survival rate [5]. Despite ongoing efforts to advance treatment in a medical imaging modality, patient with glioma still has dire prognosis rate [6–9].

DWI technique is shown to be useful in classifying gliomas tumors by grade, which was not previously viable using conventional MRI [10, 11]. DWI and ADC maps provide valuable physiological information complement to anatomical information gathered from conventional MRI. Prediction and discrimination between LGG and HGG could improve the diagnosis of patients with glioma [12, 13]. ADC images generated from standard *b* value could not distinguish between LGG and HGG at 1.5 T MR [14, 15].

The high *b* value provides better differentiation between benign and malignant brain tumors and shows the better delineation of ischemic territory in the case of acute cerebral ischemia and CNS lymphoma [16–19]. Moreover, it maximizes the contrast visualization between the lesion and normal tissue in cases of Alzheimer's disease and decreases the limitations of DWI [20, 21]. Early detection of glioma grade based on the DWI procedure considered noninvasive promising tools in the evaluation of glioma grades and could be helpful in the assessment of patient prognosis and response to therapy [22].

## 2. Materials and Methods

In the current study, an analytical comparative cross-sectional study was used to collect eligibility cases. The study population includes all suspected patients having cerebral glioma based on CT radiological findings or clinical history. Based on the MRI archive of Al-Shifa Medical Complex, 40 patients underwent brain MRI with suspected glioma from the 1st of January 2019 to the 1st of January of 2020. The sample size was a consecutive nonprobability sampling for patients with gliomas. The number of confirmed cases was 23 and included in the study. After obtaining ethical and administrative approvals, data were collected from March 2019 to March 2020.

### 2.1. MRI Data Acquisition

All patients underwent MRI procedures on a 1.5 T scanner (Magnetom Aera; Siemens Medical Solution, Erlangen, Germany) with a 16-channel head coil. The system was provided with the high-performance gradient coil 45 mT/m and the maximum slew rate of 125 mT/m/s. A routine tumor protocol was used and included axial T2 fluid attenuation inversion recovery (FLAIR) TR/TE (8400/120 ms), T2WI fast SE (TR/TE 3200/100 ms), and pre- and postcontrast (gadolinium-DTPA, Magnevist, Bayer Pharma, Berlin, Germany) orthogonal T1W spin-echo (SE) (TR/TE = 450/9 ms). The DWI sequence was obtained using echo planer imaging with standard (*b* = 1000 s/mm^2^) and high (*b* = 3000 s/mm^2^) *b* values.

The MR techniques were conducted based on the following parameters:

- TR/TE = 5000/142 ms for *b* = 1000 mm^2^/s
- TR/TE = 7300/156 ms for *b* = 3000 mm^2^/s
- Scan time = 1 : 32 min for *b* = 1000 mm^2^/s and 2 : 13 min for *b* = 3000 mm^2^/s

In addition, section thickness = 5 mm, slice gab = 1 mm, field of view = 240 × 240 mm, and matrix = 190 × 160 mm.

### 2.2. Quantitative Analysis

**Figure 1:**
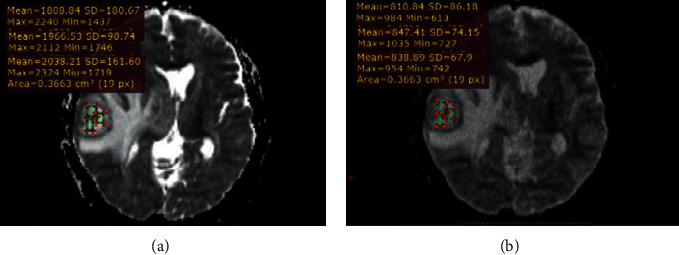
RadiAnt DICOM viewer ROI measurement calculation. This representative case shows how the three ROIs were selected manually away from edema and how the RadiAnt DICOM viewer measures the mean, maximum, and minimum automatically for each ROI on ADC_1000_ (a) and copied ROIs on identical slice position in ADC_3000_ (b).

All measurements were performed by using the RadiAnt DICOM viewer (version, 2020.1). The ROIs were manually drawn by two expert radiologists on axial 2D DWI slice that represents the majority of the solid part of the tumor. The delineation of tumor boundaries was done on an identical slice section on contrast enhancement T1WI away from either edema or necrotic regions (Figure 1).

All diffusion weight images were analyzed, and ADC maps were acquired at both *b* = 1000 and *b* = 3000 mm2/s. Two groups of ROIs were drawn on both ADC1000 and ADC3000 for each patient by an experienced radiologist. The first group includes three ROIs which were drawn at different consecutive slice sections from solid lesion to minimize the selection bias, and the second group contains three ROIs on the normal-appearing white matter (NAWM) in the contralateral side which were also taken. Tumor ROI measurements are obtained from the solid components of the tumor avoiding the measurement from cystic changes, necrosis, or even hemorrhage that may influence the ADC values [23–25].

Tumor ROI was placed regarding the contrast enhancement lesion on the axial T1WI. In contrast, ROI is placed over the most restricted area on the ADC map for nonenhancing lesions, as illustrated in Figure 1. Repeatedly, the ROI was copied to ADC1000 and ADC3000 for identical locations. The researchers used three small ROIs ranging from 0.30 to 0.50 mm^2^, and some of the conflicting results are attributed to how ROIs are placed carefully excluding cystic or necrotic parts. Kamael found the ADC values were correlated with necrosis that often occupies a large portion of HGG that influences the efficacy of grading of glioma by ADC map [26]. The ADC mean within the tumor was calculated as the average of three ADC values within the tumor. The maximum and minimum ADC values within the tumor were defined as ADC max and ADC min respectively. The ADC ratio is obtained by dividing ADC mean within the tumor by the ADC mean of contralateral NAWM as shown in Figure 1.

### 2.3. Statistical Analysis

The statistical analyses were performed using the statistical software package (MedCalc, version 19.0.4). The correlations between ADC values at both *b* values and histopathology results were investigated using the Spearman correlation analysis. Kappa-test was used to measure the agreement between ADC values for both *b* values and histopathology results. The receiver operating curve (ROC) was used to calculate the sensitivity, specificity, area under the curve (AUC), and accuracy and generate cutoff points of ADC value for both *b* values DWI.

## 3. Results

The current results revealed that out of 23 examined cases, there are 11 males and 12 females with a mean age of 37.8 ± 23 years (range: 4–78 years). The majority of cases 16 (69.6%) were less than 40 years old, and the rest is more than 50 years old (Table 1). According to histopathological results, two patients had grade II oligodendroglioma, two patients had grade II astrocytoma, three patients had grade II polymorphic xanthoastrocytoma, two patients had grade III anaplastic oligodendroglioma, and 14 of them had grade IV glioblastoma multiforme. The results revealed that seizures are the most common symptoms in glioma patients (39.1%). Coma and cognitive disorders rank the second clinical manifestation of glioma among patients (17.4%). Also, general weakness is a common clinical manifestation among 13% of glioma patients, while the rest of patients' symptoms were vertigo and memory loss. Regarding the location of the gliomas, the results revealed that gliomas in the temporoparietal lobe accounted for 34.8%, frontal lobe for 17.4%, parietal, temporal, and infratentorial lobe for 13%, respectively, and occipital lobe for 8.8% of the cases.

Based on the World Health Organization (WHO), two cases had grade I, five cases had grade II astrocytoma, diagnosed as LGG, two cases had grade III oligodendroglioma, and 14 cases had glioblastoma multiform and diagnosed as HGG. The distribution of grades, gender, and age is clarified in Table 2. The MRI procedures were performed two to three days before surgery. An expert in histopathology who has 27 years of experience defined the tumor grade through resection biopsy.

### 3.1. ADC Value at Two Different *b* Values and Glioma Grades

The ADC values of ADC mean, ADC max, ADC min, and ADC ratio values of grade II, III, and IV gliomas are summarized in Table 3. The ADC min values ranged between 0.82 ± 0.07 × 10^−3^ mm2/s and 1.13 ± 0.07 × 10^−3^ mm^2^/s at standard *b* value (*b* = 1000 mm^2^/s) and 0.48 ± 0.07 × 10^−3^ mm^2^/s and 0.76 ± 0.07 × 10^−3^ mm^2^/s at high *b* value (*b* = 3000 mm^2^/s). In measurements using *b* = 1000 and *b* = 3000, the ADC values decreased while the grade of glioma increased. Moreover, ADC mean, ADC ratio, ADC max, and ADC min values were calculated and showed that ADC values also decreased with increasing of *b* value.

### 3.2. Correlation between ADC Min Values and Histopathology Results

**Figure 2:**
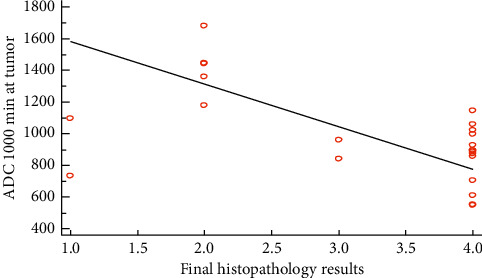
Spearman's correlation between histopathology results and ADC _1000_.

**Figure 3:**
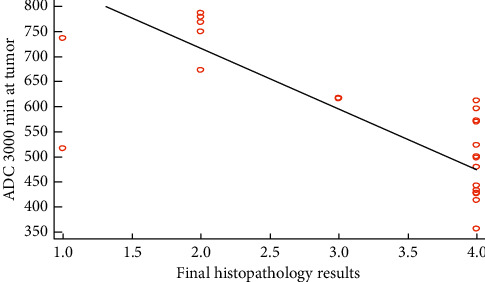
Spearman's correlation between histopathology results and ADC_3000._

Spearman's correlations for both standard and high *b* values against histopathology results were shown in Figures 2 and 3. Spearman's correlation showed a significant negative correlation between the level of significance (*r* = −0.536, *P* value = 0.008) at standard *b* value.

Spearman's correlation between ADC_min 3000_ and histopathology grading results was of high statistical significance (*r* = −0.722, *P* ≤ 0.001).

### 3.3. Qualitative Results of ROC Analysis and ADCs' Values for Tumor Grading

**Figure 4:**
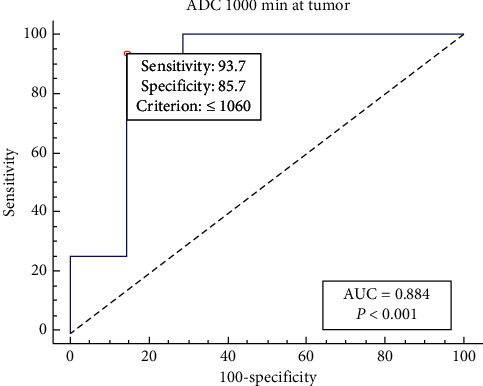
ROC curves for ADC_min_ values at *b* values of 1000 mm^2^/s.

**Figure 5:**
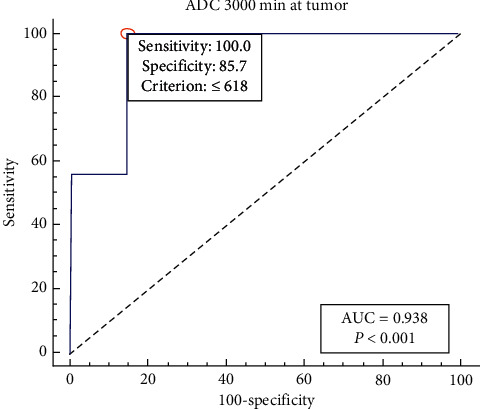
ROC curves for ADC_min_ values at *b* = 3000 mm^2^/s.

ROC analysis was conducted to generate appropriate cutoff points and corresponding sensitivity, specificity, Youden index, and AUC. The cutoff values of ADC _min_ at *b* values of 1000 and 3000 mm^2^/s were 1.6 × 10^−3^ mm^2^/s and 0.618 × 10^−3^ mm^2^/s, respectively. Sensitivity and specificity were higher for ADC_min_ values at high *b* value compared to standard *b* value (Table 4, Figures 4 and 5).

### 3.4. Agreement between ADC Min at Standard and High *b* Values and Histopathology Findings

A stronger agreement was found between ADC 3000 and histopathology results compared with ADC1000 (*k* = 0.893, 0.794) as illustrated in Table 5.

**Figure 6:**
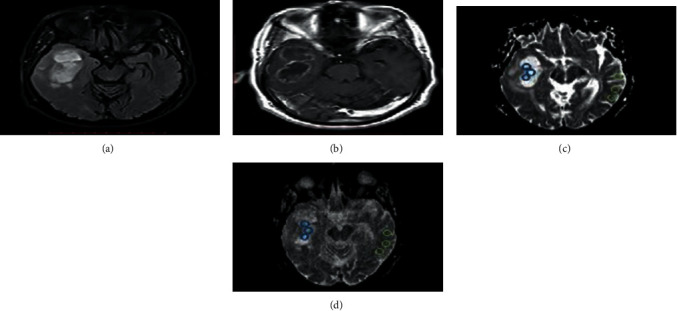
49-year-old man with GBM in the right temporal lobe. Based on the signal characteristics of T2WI and postcontrast T1WI, three ROIs were placed on the solid component of the tumor and three ROIs were placed on the contralateral NAWM on ADC images. Images (c, d). (a). On the T2 FLAIR sequence (TR/TE = 8800/120 ms), the GBM is hyperintense. (b). On T1WI SE (TR/TE = 410/9 ms) after contrast injection, the tumor is hypointense with ring enhancement. (c). ADC map at standard *b* value (TR/TE = 6100/152 ms; *b* = 1000s/mm^2^). (d). ADC map at high *b* value (TR/TE = 8600/152 ms; *b* = 3000s/mm^2^). The tumor is more hypointense on the ADC map obtained at a high *b* value than the ADC map obtained at standard *b* value.

**Figure 7:**
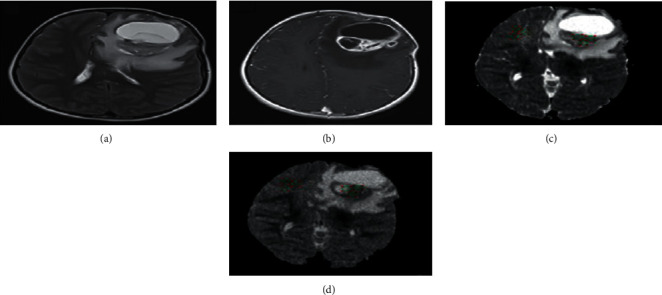
12-year-old female patient with astroblastoma (grade II) in the left frontoparietal lobe. Based on the signal characteristics of T2WI and postcontrast T1WI, three ROIs were placed on the solid component of the tumor and three ROIs were placed on the contralateral NAWM on ADC images. Images (c, d). (a). On the T2 FLAIR sequence (TR/TE = 8800/120 ms), the tumor is hyperintense. (b). On T1WI SE (TR/TE = 410/9 ms) after contrast injection, the tumor is hypointense with ring enhancement. (c). ADC map at a standard *b* value (TR/TE = 6100/152 ms; *b* = 1000s/mm^2^). (d). ADC map at a high *b* value (TR/TE = 8600/152 ms; *b* = 3000s/mm^2^). The tumor is more hypointense on the ADC map obtained at a high *b* value than the ADC map obtained at a standard *b* value.

Representative cases are shown in Figures 6 and 7.

## 4. Discussion

The study was designed to investigate the accuracy of DWI at both high and standard *b* values (*b* = 1000s/mm^2^ and *b* = 3000 s/mm^2^) with 1.5 Tesla MRI system and to examine its ability in distinguishing LGG from HGG in clinical practice based on histological grades finding. Manipulation of diffusion parameters like duration, strength, and diffusion sensitivity can alter the image contrast [27]. MR technology has upgraded and improved DWI with *b* values up to 10,000. Although *b* = 1000 is remarkably useful in the detection and delineation of restricted diffusion in clinical practice, high *b* value is critical in future assessment and investigation. DWI acts as a biomarker of free water diffusion measurements and shows an increase in cellularity with high tumor grade. Several studies focused on using high *b* value in the grading of glioma and suggest its effectiveness with increased sensitivity and specificity in glioma grading compared with standard *b* value [28–30]. The results confirmed that the ADC_3000_ is more helpful than ADC_1000_ in the grading of glioma. The best cutoff point for distinguishing LGG from HGG was the ADC_min_ value obtained at a high *b* value.

Doskaliyev et al. reported that the ADC values were inversely correlated with tumor cellularity, and these statistical differences were more pronounced at high *b* value (*b* = 4000 s/mm^2^) compared with standard *b* value (*b* = 1000 s/mm^2^) [31]. Chen et al. have also demonstrated an inverse correlation between tumor cellularity and ADC values of glioma [32]. Alvarez-Linera et al. have found that the ADC values for HGG were significantly lower than those for LGG at standard and high *b* values, and HGG tended to have high signal intensity (SI) relative to contralateral NAWM, and high *b* value was more sensitive and specific in the differentiation between LGG and HGG [33]. Yamasaki et al. reported that the high *b* value reflects more tissue diffusivity than the standard *b* value [34]. The study results attributed to increasing tumor cellularity that reflects lower ADC value and subsequently HGG.

High *b* value DWI is useful in the grading of gliomas and more effective than standard *b* value in distinguishing between pseudo and true responses in patients with recurrent glioma after bevacizumab treatment [34]. In addition, high *b* value was useful in the diagnosis of acute infarction and white matter degeneration in Alzheimer's disease in addition to the differentiation between malignant lymphoma and glioblastoma [20, 31, 35]. DWI acquired at a high *b* value has more conspicuous hyperintensity in HGG and hypointensity in LGG than standard *b* value DWI [28]. Kang et al. explored the role of histogram analysis for standard and high *b* value based on the entire tumor volume and the study emphasized that ADC_min_ for both ADC_1000_ and ADC _3000_ decreases with increasing tumor grade for tumor grades II, III, and IV, and a statistical difference was found between three grades regarding ADC_min_ [36]. In contrast, the study results imply that a DWI at *b* = 1000 is not high enough to discriminate between LGG and HGG.

Higher magnetic field strength and powerful gradient coil may permit higher *b* value and more diffusion sensitivity that facilitate the differentiation between LGG and HGG. In this study, the ADC _min_ at *b* = 3000 achieved the lowest degree of overlapping and confirmed the previous results that the high *b* value gives more reliable results. Hu et al. explored the efficacy of 12 different *b* values ranging from 500 to 4500 mm^2^/s in the discrimination between LGG and HGG and reported that the signal of tumor tissue in LGG decreases rapidly with an increase of *b* value [37]. When the *b* value shifted from 1000 mm^2^/s to 3000 mm^2^/s, the ADC values decrease approximately by 30%–35% for the same ROIs [38]. This phenomenon can explain biexponential signal intensity decay and fast and slow diffusion, which actually corresponds to extra- and intracellular space, respectively [27]. The fast component diffusion signal intensity is governed by a low *b* value, whereas slow component diffusion signal intensity is dominated by a high *b* value [39–41]. In this study, ADC parameters were derived only from the solid portion of the tumor at 1.5 T, and unlike Cihangiroglu et al., we did not find statistical differences between glioma grades III and IV at ADC_min_ obtained at high *b* value. [22].

The study results confirmed that the ADC_min_ value was able to distinguish LGG from HGG most accurately among all ADC values. These results agree with several studies that had studied the minimum ADC extensively [42–44]. Considering histopathological results as the gold standard, ROC analysis reveals that the high *b* value can distinguish LGG from HGG with better sensitivity and specificity (100%, 85.7%) than standard *b* value DWI with 93.7% and 85.7%, respectively. According to cellularity, the cutoff point that is able to distinguish LGG from LGG is equal to 1.06 × 10^−3^ mm^2^/s. Thus, the ADC value equal to or smaller than this value can be recognized as HGG, while the ADC values that are higher than this value are considered as LGG. The current results agree with Murakami et al.'s study that determines that the cutoff point at ADC_min_ 1000 was 1.01 × 10^−3^ mm^2^/s [25]. The threshold of ADC_min_ that could separate LGG from HGG was 1.48 × 10^−3^ mm^2^/s LGG [44]. Hu et al. reported that the cutoff point at ADC_1000_ for the differentiation between LGG and HGG was 1.11 × 10^−3^ mm^2^/s, and AUC was 0.905, sensitivity was 82.7%, and specificity was 85.2% [37]. Nearly, the same results were reported by Hilario et al. and revealed that the ADC threshold value for glioma grading was 1.185 × 10^−3^ mm^2^/s, and sensitivity and specificity were 97.6% and 53.1%, respectively [43].

A high *b* value can more effectively grade glioma compared with ADC value based on standard *b* value and revealed that the cutoff point at a high *b* value is very close to the study results which equals 0.634 ± 0.15 ×10^−3^ mm^2^/s with sensitivity and specificity of 92.3% and 92.3%, respectively, and an accuracy of 94.8% which is consistent with the study results [19]. Cihangiroglu et al. (2017) also reported that the cutoff point at high *b* value equals 0.62 ×10^−3^ mm^2^/s with a sensitivity of 80%, a specificity of 81.8%, and an accuracy of 62% [22]. Zeng et al. and Han et al. reported slightly higher cutoff points of 0.890 × 10^−3^ mm^2^/s and 0.875 × 10^−3^ mm^2^/s, respectively [30, 37]. Hu and his colleagues investigated the efficacy of high *b* value in the discrimination between LGG and HGG, and the results confirmed that high *b* value achieved high sensitivity compared with standard *b* value (85.7% and 82.7%), respectively, and the cutoff point at ADC_3000_ was 0.763 × 10^−3^mm^2^/s with an AUC of 0.897, a sensitivity of 85.7%, and a specificity of 81.2% [37]. The current study showed that the high *b* value achieved a higher agreement and was more valuable in the prediction of a histological grade than the standard *b* value (*k*= 0.89 and 0.79, respectively). In this study, the selection of high *b* value (*b* = 3000 s/mm^2^) is for two reasons. First, higher *b* value may accentuate the anisotropic effect, and this diminishes the utility of high *b* value DWI in areas where the white matter tracts are more prominent [45]. Secondly, increasing the *b* value increases the time of scanning, the signal-to-noise ratio (SNR) becomes worse, and the image gets more likely to be exposed to patient-related motion artifact [46]. Although the ADC_min_ at *b* = 3000 was inversely correlated with histological grades of gliomas, there is some overlapping between grades. Therefore, it is mandatory to evaluate the glioma grade on high *b* value DWI complementary to SI of other MRI routine sequences. The main limitations of this study are the small sample size that represents the biggest obstacle that faced us, the delay time of getting histopathology results, and the referral of many cases of suspected glioma to hospitals outside the Gaza Strip. Another limitation is methodological challenges where all measurements were gained regarding the DWI axial 2D sequence, not 3D, because the 3D DWI sequence requires more scan time and the possibility of motion artifacts increases.

## 5. Conclusion

The ADC _min_ values were negatively correlated with glioma grades, and the correlation was more discernible at the high *b* value that may be useful in the prediction of glioma grading. According to the results of ROC analysis, ADC parameters derived from a high *b* value DWI might be more powerful than those estimated from a standard *b* value DWI. In addition, a high *b* value DWI attained higher agreement than the standard *b* value DWI when compared to histopathological findings. High *b* values provide an opportunity to gain insight as a simple and effective tool in glioma grading and potentially improve patient outcomes through accurate early noninvasive diagnosis, aiding tumor characterization, and facilitating early treatment planning. The integration of the DWI map into clinical practice could assist in better management decisions and treatment.

## Figures and Tables

**Table 1:** Distribution of demographic and related tumor characteristics of cases.

Variables, *n* = 23	Frequency	Percentage (%)
*Gender*		
Male	11	47.8
Female	12	52.2

*Age*		
Less than 30 y	8	34.8
From 30 to 50 y	8	34.8
More than 50 y	7	30.4

*Histopathology types*		
Oligodendroglioma	2	8.70
Astrocytoma	2	8.70
Polymorphic xanthoastrocytoma	3	13.0
Anaplastic oligodendroglioma	2	8.70
Glioblastoma multiforme	14	60.9

*Tumor location*		
Frontal	4	17.4
Parietal	3	13.0
Temporal	3	13.0
Occipital	2	8.80
Tempo-parietal	8	34.8
Infratentorial	3	13.0

*Symptoms*		
Vertigo	2	8.70
Coma	4	17.4
Seizures	9	39.1
Memory loss	1	4.30
Weakness	3	13.0
Abnormal behavior	4	17.4

**Table 2:** Distribution of grade, age, and gender within the study sample.

	Patient *n* (%)	Age (mean ± SD)	Gender (F/M)
Grade 1	2 (8.7)	6.5 ± .7 years	1/1
Grade 2	5 (21.7)	14.4 ± 5.5 years	4/1
Grade 3	2 (8.7)	44.5 ± 14 years	1/1
Grade 4	14 (60.9)	49 ± 19 years	6/8

**Table 3:** ADC_mean_, ADC_ratio_, ADC_max_, and ADC_min_ for grade II, III, and IV gliomas for two different *b* values.

ADC value	*b* values (s/mm2)	G2	G3	G4
ADC _mean_	1000	1.40 ± 0.22	1.22 ± 0.19	1.09 ± 0.22
	3000	0.95 ± 0.09	0.80 ± 0.82	0.68 ± 0.08

ADC _ratio_	1000	2.10 ± 1.39	2.00 ± 0.45	1.40 ± 0.37
	3000	1.50 ± 0.20	1.40 ± 0.12	1.30 ± 0.39

ADC _max_	1000	1.79 ± 0.30	1.73 ± 0.64	1.38 ± 0.28
	3000	1.17 ± 0.15	1.04 ± 0.24	0.90 ± 0.11

ADC _min_	1000	1.13 ± 0.17	0.89 ± 0.85	0.82 ± 0.17
	3000	0.76 ± 0.07	0.61 ± 0.01	0.48 ± 0.07

**Table 4:** Diagnostic accuracy in distinguish LGG from HGG based on ADCmin values.

Variables, *n* = 23	AUC	Sensitivity (%)	Specificity (%)	+PV	−PV	Cutoff value	*P* value	Youden index
ADC 1000	884	93.75	85.7	93.7	85.7	≤1060	<.001	0.7946
ADC 3000	938	100	85.7	94.1	100	≤618	<.001	0.8571

**Table 5:** Agreements between ADC values for both *b* values and histopathology results.

	Kappa	*P* value
ADC1000	0.794	<0.001^*∗*^
ADC3000	0.893	<0.001^*∗*^

## Data Availability

The data used to support the findings of this study are available from the corresponding author upon request.
